# Left Atrial Enhancement Correlates With Myocardial Conduction Velocity in Patients With Persistent Atrial Fibrillation

**DOI:** 10.3389/fphys.2020.570203

**Published:** 2020-11-12

**Authors:** Rheeda L. Ali, Norman A. Qureshi, Silvia Liverani, Caroline H. Roney, Steven Kim, P. Boon Lim, Jennifer H. Tweedy, Chris D. Cantwell, Nicholas S. Peters

**Affiliations:** ^1^ElectroCardioMaths Programme of The Imperial Centre for Cardiac Engineering, Imperial College London, London, United Kingdom; ^2^National Heart & Lung Institute, Imperial College London, London, United Kingdom; ^3^Department of Bioengineering, Imperial College London, London, United Kingdom; ^4^School of Mathematical Sciences, Queen Mary University of London, London, United Kingdom; ^5^Abbot Medical, St. Paul, MN, United States; ^6^Department of Aeronautics, Imperial College London, London, United Kingdom; ^7^School of Biomedical Engineering and Imaging Sciences, King’s College London, London, United Kingdom

**Keywords:** LGE-MRI, image segmentation, fibrosis, electro anatomical mapping, conduction velocities, Atrial fibrillation, left atrium

## Abstract

**Background:**

Conduction velocity (CV) heterogeneity and myocardial fibrosis both promote re-entry, but the relationship between fibrosis as determined by left atrial (LA) late-gadolinium enhanced cardiac magnetic resonance imaging (LGE-CMRI) and CV remains uncertain.

**Objective:**

Although average CV has been shown to correlate with regional LGE-CMRI in patients with persistent AF, we test the hypothesis that a localized relationship exists to underpin LGE-CMRI as a minimally invasive tool to map myocardial conduction properties for risk stratification and treatment guidance.

**Method:**

3D LA electroanatomic maps during LA pacing were acquired from eight patients with persistent AF following electrical cardioversion. Local CVs were computed using triads of concurrently acquired electrograms and were co-registered to allow correlation with LA wall intensities obtained from LGE-CMRI, quantified using normalized intensity (NI) and image intensity ratio (IIR). Association was evaluated using multilevel linear regression.

**Results:**

An association between CV and LGE-CMRI intensity was observed at scales comparable to the size of a mapping electrode: −0.11 m/s per unit increase in NI (*P* < 0.001) and −0.96 m/s per unit increase in IIR (*P* < 0.001). The magnitude of this change decreased with larger measurement area. Reproducibility of the association was observed with NI, but not with IIR.

**Conclusion:**

At clinically relevant spatial scales, comparable to area of a mapping catheter electrode, LGE-CMRI correlates with CV. Measurement scale is important in accurately quantifying the association of CV and LGE-CMRI intensity. Importantly, NI, but not IIR, accounts for changes in the dynamic range of CMRI and enables quantitative reproducibility of the association.

## Introduction

Success rates of catheter ablation for persistent AF is hindered by our poor understanding of the underlying mechanisms of AF persistence. Central to improving this understanding is the relationship between local myocardial conduction properties and the underlying tissue architecture, determined clinically by estimating myocardial fibrotic burden using late-gadolinium enhanced cardiac magnetic resonance imaging (LGE-CMRI). The challenges to determining this relationship are in part due to limitations and inconsistencies in the acquisition, interpretation and the registration of high-resolution imaging and electroanatomic mapping (EAM) data to allow correlative analyses.

It has previously been established that an electro-architectural relationship exists between myocardial fibrosis and CV on a regional and whole-heart level (Badger et al., 2009; Fukumoto et al., 2016). However, if such a relationship exists on a localized level, LGE-CMRI may fulfill its potential as a non-invasive tool to map myocardial conduction properties for risk stratification and treatment guidance.

Current 3D EAM systems with high-density multi-electrode contact mapping catheters can provide detailed spatio-temporal information on the functional behavior of the endocardium. Local CV can give a clear interpretation of underlying tissue health and identify the presence of non-conducting fibrotic tissue through the analysis of wave-front propagation patterns (Spach and Kootsey, 1983; Tanaka et al., 2007). The accurate evaluation of local CV requires invasive contact mapping with subsequent laborious post-processing of acquired electrograms.

Late-gadolinium enhanced cardiac magnetic resonance imaging is a well-established non-invasive technique to visualize myocardial fibrosis and has been corroborated with histomorphometric validation (Schmidt et al., 2007). Fibrotic atrial imaging has had mixed success due to the current limits of MRI resolution, the patchy nature of atrial fibrosis and difficulties in inter-patient scar-thresholding. As a consequence, several post-processing algorithms and intensity normalization approaches have been developed to improve the robustness of LA fibrosis-mapping from LGE-CMRI (Pontecorboli et al., 2017). Although the extent of enhancement has been associated with conventional markers of atrial structural remodeling such as LA dimension (Habibi et al., 2015), and clinical outcomes following catheter ablation (McGann et al., 2011), there is continued uncertainty surrounding the exact nature and pathological state of the atrial myocardium delineated by high-intensity regions.

We sought to test the hypothesis that a systematic and objective approach to the acquisition and spatial correlation of CV and LGE-CMRI data can define a reproducible electro-architectural relationship at clinically relevant scales.

## Materials and Methods

**FIGURE 1:**
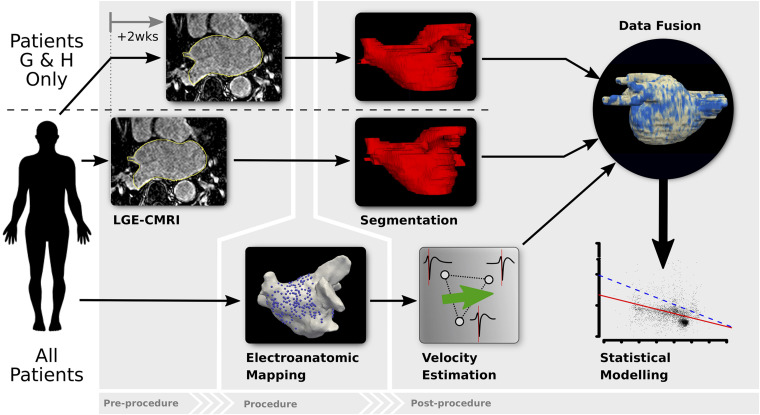
Diagram of the analysis methodology. All patients undergo LGE-CMRI prior to the ablation procedure (patients G and H had 2 pre-procedural LGE-CMRI to assess reproducibility of LA fibrosis-mapping). Prior to any ablation, LA EAM during pacing is performed. Localized CV is estimated from EAM data and LGE-CMRI images are segmented to estimate fibrosis density. Data is co-registered and fused, after which statistical modeling is undertaken.

A diagram showing the key steps of the data collection and analysis methodology used in the study is shown in Figure 1.

### Study Population

Patients with symptomatic persistent AF (based on the classification of AF by published guidelines from the AHA/ACC/HRS/ESC) presenting for their first ablation to Imperial College Healthcare NHS Trust were prospectively enrolled. The study was approved by the Local Research and Ethics Committee for Imperial College Healthcare NHS Trust and written informed consent was obtained from each patient. Patients with contraindications to undergoing LGE-CMRI were excluded from the study.

### Data Acquisition

Each patient underwent LGE-CMRI prior to the ablation procedure. MRI acquisition was performed using a 1.5T Philips Achieva MR system, and a 5- or 32-element phased-array cardiac coil. LGE-CMRI was performed in the axial orientation 12–20 min following the 20 ml bolus of gadobenate dimeglumine contrast agent, using an ECG triggered, free-breathing navigator-gated whole heart 3D spoiled gradient echo acquisition sequence. Resolution was at 1.5 × 1.5 × 4 mm and reconstructed to 1.25 × 1.25 × 2 mm. Complete LA coverage was obtained with 40–50 slices. Data were acquired within a window of 100–150 ms within each R-R interval depending on heart rates, with a low-high k-space ordering and spectral pre-saturation with inversion recovery (SPIR) for fat suppression. The inversion recovery delay was determined from a Look-Locker sequence, with an inversion time chosen to null myocardial signal. MRI scans were performed in patients in rate-controlled AF. To assess the robustness and reproducibility of the methodology, two patients (denoted throughout as patients G and H) underwent two pre-ablation LGE-CMRI scans, 2 weeks apart.

Catheter ablation was performed within 2 weeks from the LGE-CMRI scan. All anti-arrhythmic drugs were discontinued for at least 5 half-lives, and amiodarone was discontinued at least 60 days prior to the ablation procedure. All procedures were performed in the post-absorptive state under general anesthesia. Transesophageal echocardiography was performed in all patients once anesthetized to exclude LA appendage clot, and to subsequently guide transseptal puncture. A deflectable decapolar catheter (Inquiry^TM^, St Jude Medical, St. Paul, MN, United States) was positioned in the coronary sinus to record electrograms, pace the atrium, and serve as a temporal reference. Single *trans*-septal punctures were performed using a Brokenbrough needle through a fixed curve long-sheath (SL0, St Jude Medical, MN, United States). Unfractionated heparin was administered to achieve an activated clotting time of 300–350 s throughout the procedure.

An impedance-based EAM system (NavX EnsiteTM Velocity, St Jude Medical, MN, United States) was used. The LA geometry and all subsequent data were acquired using a 20-pole (1 mm electrodes) double-loop catheter (Inquiry^TM^ AFocusII^TM^, St Jude Medical, MN, United States) with 4 mm electrode spacing. Before each acquisition, the AFocusII catheter was held tangentially to the endocardial surface, enabling stable tissue contact. Patients presenting in AF underwent external DCCV before any mapping was conducted. Following the acquisition of the LA geometry, high-density LA activation mapping was performed. The left atrium was paced from one of more sites [i.e., the coronary sinus, roof of left atrium and left atrial (LA) appendage] at pacing rates of 250, 300, 350, and 600 ms. The pacing protocol included a drive train of 8 beats to ensure that LA capture and activation was consistent (avoiding latency and decrementation), and also to facilitate the stability of AFocusII catheter at the particular site of the left atrium that was being interrogated/mapped.

**FIGURE 2:**
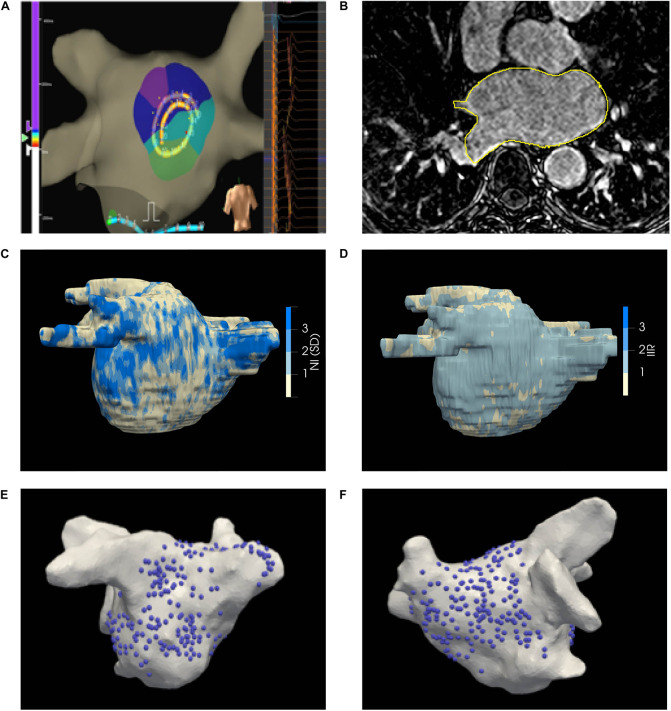
**(A)** Ensite^TM^ Velocity mapping system used for collecting endocardial electrograms. The LA was paced over a range of CLs and the acquired unipolar electrograms are shown in the right panel. **(B)** LGE-CMRI axial slice with manual segmentation delineated in yellow **(C)** Illustrative patient-specific map NI. **(D)** Illustrative patient-specific map of IIR for same patient as **(C, E, F)** Distribution of electrograms on the posterior and anterior walls of the LA, respectively.

The LA was paced using a drive train protocol of 8 beats from the coronary sinus, roof, and/or LA appendage. Unipolar electrograms were recorded and displayed at filter settings of 0.5–100 Hz during the procedure, where the 20 recordings together form a kernel as shown in Figure 2A. The electrode positions were projected to the geometry by the EAM system (Figures 2E,F). Electrodes more than 5 mm away from the surface were disregarded.

Data were acquired at multiple locations on the LA, focused mainly on the posterior endocardial wall where the highest propensity of fibrosis was expected to be found (Cochet et al., 2015). Local activation times (LATs) were calculated by the EAM system relative to a reference electrode and assigned as the time of the maximum negative gradient of the unipolar electrogram. Electrograms were assessed post-procedurally by an experienced Cardiac Electrophysiologist and those indicative of poor contact or high noise were rejected. Following mapping, pulmonary vein isolation was achieved. All patients were observed for a further 24 h prior to discharge. No complications were observed in this cohort of patients.

### MRI/EAM Segmentation and Registration

The LA epicardial surface on LGE-CMRI images was manually segmented by an experienced Cardiac Radiologist, as shown in Figure 2B, and the epicardial surface was extracted. The EAM surface was co-registered to the MRI surface (Rueckert et al., 1999; Studholme et al., 1999; Schnabel et al., 2001; Ali et al., 2015). The accuracy of the registration process was estimated by target registration error (Fitzpatrick and West, 2001). Projected EAM surface electrode positions were mapped under the computed surface transformation to the MRI surface. The operators performing the ablation procedure were blinded to the generated LA scar-maps derived from the LGE-CMRI.

### Local and Regional Conduction Velocity Estimation

Conduction velocity (CV) was estimated both locally and regionally. Regional CV was estimated by fitting a model of an ideal circular propagating wavefront to the positions and LATs of the 20 electrodes of a given kernel. Additionally, the wavefront radius, r, and residual error, η, of the fit were calculated (Roney et al., 2014). High η indicate that the wavefront is not sufficiently smooth within the kernel. Kernels with η < 5 s/mm were rejected as the wavefront violated the planarity assumptions required by the local CV analysis below.

The local CV is calculated using the principle of triangulation which uses the differences in LATs across unique groups of 3 mapping electrodes (triads) and their interelectrode distance (Kojodjojo et al., 2005). This approach provides accurate estimates of velocities in areas of just a few mm (Badger et al., 2009), but assumes planar propagating wavefronts (Cantwell et al., 2014). Triads were only chosen between concurrent recordings within the same kernel to avoid any inter-beat variability of wavefront propagation. The minimum interelectrode distance between any pair of electrodes in a triad was constrained to be greater than the registration error.

### Detection and Evaluation of Left Atrial Wall Intensities From LGE-CMRI

Raw absolute LA wall image intensities were extracted from the LGE-CMRI image as the maximum voxel intensity along a 3 mm inward-facing normal from the segmented epicardial surface.

Left atrial wall LGE-CMRI intensities are acquired in arbitrary units and their average brightness and dynamic range varies between images, even between multiple scans of the same subject. Local gadolinium uptake was quantitatively evaluated through two intensity normalization techniques: (1) Normalized intensity (NI) is calculated from the raw intensity by subtracting the mean intensity of the LA blood pool and dividing by the standard deviation (SD) of the blood pool voxels (Kolipaka et al., 2005; Fukumoto et al., 2016) Image Intensity Ratio (IIR) is calculated as the ratio of raw intensity values and the mean intensity of the LA blood pool (Khurram et al., 2014). Both these metrics convert the raw intensities to quantities which can be compared, and are routinely utilized in LA scar-mapping with LGE-CMRI (McGann et al., 2011; Khurram et al., 2014; Fukumoto et al., 2016). The mean and SD of the blood pool were calculated by shrinking the segmented epicardial surface by 5 voxels (3 mm). The average NI or IIR value on the area enclosed by each triad of transformed electrode positions was then calculated (Ali et al., 2014). A representative map of NI on the segmented surface is shown in Figure 2C.

### Reproducibility

A sub-group of two patients had two pre-ablation LGE-CMRI scans, separated by 2 weeks. Segmentation, registration and construction of the LA scar map were performed independently on each image. A single EAM dataset was used per patient for determining association. The data from each scan were included in the statistical analysis as separate datasets. If the image processing and registration methodology is reproducible, the estimated association should not be significantly different between the two scans of the same patient.

### Statistical Analysis

Continuous variables are given as mean ± SD; categorical variables are given as percentages. A linear mixed-effects model was used to characterize the relationship between LA wall intensity, using either NI or IIR, and CV. Likelihood-ratio tests were used to compare if models, fit by maximum likelihood, were significantly different. The inclusion of CL did not significantly improve the model fit and was excluded from the final model.

A multilevel model was used to characterize the relationship between LA wall intensity and CV. Multilevel models provide a mechanism to account for, and quantify, variation in model intercepts and slopes across patients and cycle lengths. The association between normalized intensity, *I*, and CV, V was modeled as

$$V_{i\,j\,k}=\left(\beta_{0}+u_{j}+v_{k}\right)+\left(\beta_{1}+\beta_{2\,j}\right)\,I_{i\,j\,k}+\varepsilon_{i\,j\,k}$$

where β_*0*_ is the overall intercept, and *u*_*j*_ and *v*_*k*_ are random effects associated with patients and cycle lengths. β_*1*_ captures the effect due to intensity and is the primary parameter of interest in this study, representing the overall association between CV and intensity across all patients and cycle lengths. The β_*2j*_ values represent per-patient random slopes; that is, the patient-specific deviation from β_*1*_. Likelihood-ratio tests were used to compare if models, fit by maximum likelihood, were significantly different. The model reported here was statistically significant (*p* < 0.001) compared to all other simpler models without random intercepts or slopes. The inclusion of a random slope for cycle length did not significantly improve the model fit.

Two-sided *p*-values with *p* < 0.05 were considered to indicate statistical significance. Statistical analyses were performed using R version 3.4.3 (The R Foundation for Statistical Computing).

## Results

### Study Population

Due to the large number of data points collected per patient at multiple paced CL, a total of 8 patients provided sufficient data for the purposes of this study. A summary of the clinical characteristics is given in Table 1. Patients are denoted as A-H.

**TABLE 1:** Clinical demographics of patients.

**Patient characteristics (*n* = 8)**	
Age (y)	62 ± 11
Male	5 (62.5)
Mean LA size on TTE (mm)	41 ± 6
MeanCHADS2 score	2.4 (0–5)
Mean left ventricular EF (%)	62 ± 8
Hypertension	3 (37.5)
Diabetes mellitus	1 (12.5)
Cerebrovascular disease	1 (12.5)
Coronary artery disease	2 (25)
History of heart failure	0 (0)
Duration of persistent AF (months)	19 ± 10.7
Anti-arrhythmic drug therapy	6 (75)
(beta-blocker, flecainide, and amiodarone)	

### Data Quality

All EAM surfaces were registered to their corresponding MRI surfaces for co-localization of image intensity with CV. Average target registration errors were 3.08 mm (range 1.94–5.71 mm).

A total of 267 kernels were acquired across all patients, comprising a total of 5340 mapping points. In total 171 kernels were rejected due to non-planarity of the underlying wavefront (η < 5 s/mm). A total of 96 complete or partial kernels (mean 12 kernels/patient, range 2–13) remained. An average of 435 triads were formed per kernel (range 1–1140 triads/kernel).

### Distribution of Conduction Velocities and Left Atrial Wall Intensities

**FIGURE 3:**
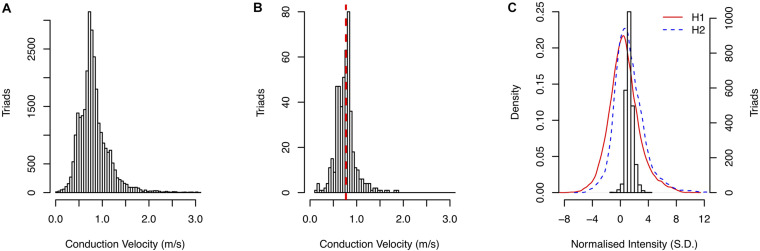
**(A)** Distribution of CV measurements in the study. **(B)** Comparison of local CV measurement distribution with regional CV estimate (red dashed line). **(C)** Distribution of sampled NI for patient H second scan and the density of NI across entire atrial surface for both scans.

An overview of the data used in this study is shown in Figure 3 and summary statistics for CV and NI for each patient are given in Supplementary Table 1. The CV sampled across all patients is given in Figure 3A. The mean CV was 0.85 m/s. A representative example of the distribution of calculated CV is shown in Figure 3B for a kernel from Patient H, paced at a cycle-length of 600 ms, with the corresponding regional CV estimate indicated by the red line. Good coherence between the local and regional algorithms was observed across all kernels in the study.

An example of the distribution of gadolinium enhancements using NI (Patient H, second scan) is shown in Figure 3C. The distributions of NI across the entire segmented atrial surface for the first and second scans are shown by the red and blue lines, respectively.

### Influence of Triad Area

**FIGURE 4:**
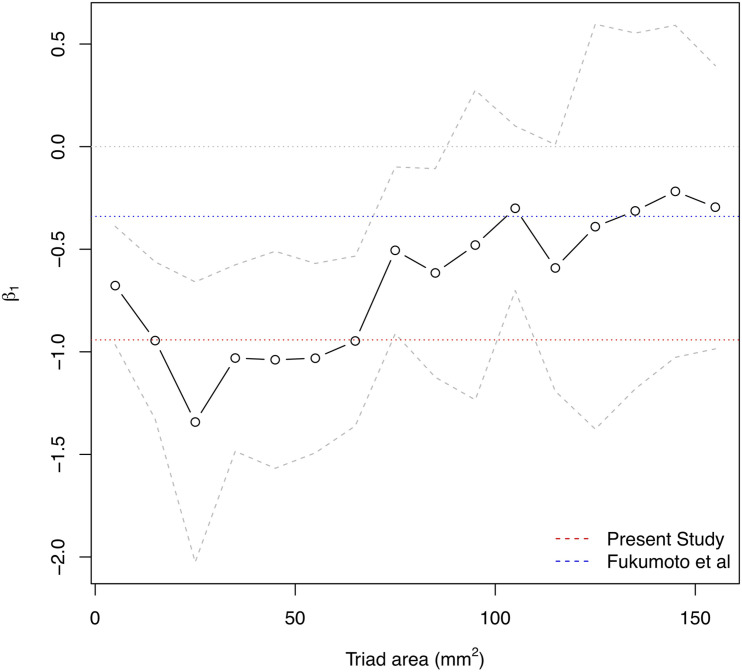
Influence of measurement area on the overall β_1_ estimate for association of CV with NI. Solid area denotes 95% confidence intervals. Larger measurement areas lead to lower magnitude β_1_ estimate. The dotted red line indicates final β_1_ estimate from present model for localized measurements with areas <80 mm^2^. The dotted blue line indicates beta estimate from Fukumoto et al^1^.

Triads were only formed within individual kernels from those electrodes in good contact with the myocardium. Triad areas ranged from 0.12 mm^2^ to 590 mm^2^. The change in CV per unit increase in LGE-CMRI intensity, denoted β_*1*_, was measured across all patients and CL. The effect of measurement area on this slope was studied by binning triads by their area and fitting the model to those triads within each bin separately. Bins were chosen as 10 mm^2^ wide non-overlapping intervals in the range 0–160 mm^2^. Beyond this range there were insufficient data to generate reliable statistical models. Figure 4 shows the change in the slope with increasing triad area. The magnitude of the slope was found to decrease with increasing area. To address the hypothesis that CV is associated with LGE-CMRI enhancement, specifically at small scales, only those triads with area <80 mm^2^ were considered for the remainder of the study.

### Association of Localized LGE Intensity With CV

**FIGURE 5:**
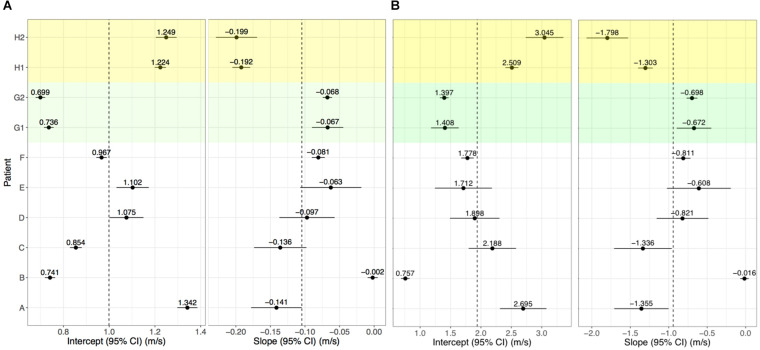
Per-patient model intercepts (left) and slopes (right) for NI **(A)** and IIR **(B)**. Values indicate the per-patient difference from the model’s overall intercept () and slope (NI: 1.00 m/s, = −0.104; IIR: = 1.94 m/s, and = −0.942. G1, G2 and H1, H2 correspond to repeat analyses of patients G and H for assessing reproducibility.

Conduction velocity correlated with LGE-CMRI intensity (slope = −0.104 m/s change in CV per unit increase of NI, *p* < 0.001). The CV at 0 S.D. (model intercept) was 1.00 m/s, which is within the expected physiological range of healthy myocardium (Ramanathan et al., 2006; Cantwell et al., 2015). Per-patient slopes and intercepts are shown in Figure 5A. Six of the per-patient slopes are significantly different from the overall slope (*p*-values < 0.05). Supplementary Figure 1 shows corresponding CV and NI at each triad for all kernels from Patient G, scan 2. The overall association and patient-specific association from the statistical model are also highlighted.

No random effect relating CL with NI was included in the model (based on the outcome of likelihood-ratio tests). Only one CL (350 ms) had an intercept significantly different from the overall intercept. Variations in the intercepts with CL were two orders of magnitude smaller than the overall intercept, suggesting there is negligible change in the relationship with respect to CL.

### Reproducibility of LGE-CV Evaluation

For Patients G and H, who underwent two LGE-CMRI scans, the images were independently segmented, registered with the clinical data and fused to examine reproducibility. These are shown as G1, G2, H1, and H2 in Figure 5A. For both patients, the slope and intercept for the first and second scans did not differ with statistical significance when using NI.

### Comparison of Left Atrial Wall Normalization (NI vs IIR)

Left atrial wall intensity was assessed using NI and IIR. The statistical model was modified to also examine the relationship between IIR with CV. This identified a change in CV of −0.942 m/s per unit increase in IIR (*p* < 0.001). The CV was estimated as 1.00 m/s at an IIR value of 1.0, which corresponds to an NI value of 0 S.D. Per-patient slopes and intercepts for the association of CV with IIR are shown in Figure 5B. As for NI, six of the patients have slopes which are significantly different from the overall slope. However, the confidence intervals are larger and reproducibility is poor, as evidenced by a significant difference in the random slopes of H1 and H2.

## Discussion

### Main Findings

We have demonstrated a localized relationship between local myocardial CV and LA wall LGE in patients with persistent AF on clinically relevant scales comparable to a mapping catheter electrode or ablation lesion. Higher normalized LA intensities represent increased structural fibrotic remodeling and this corresponds to slower CV. The overall estimate for the change in CV with each unit increase in LA intensity was found to be substantially larger in magnitude than previously reported over larger spatial scales. Triad size was found to quantitatively affect the slope, with larger area measurements reducing the magnitude of the corresponding relationship. NI was identified to be a more effective intra- and inter-patient measure of LA intensity normalization compared to IIR, leading to increased confidence in the estimate of and improved intra-patient reproducibility. There were no significant differences in the relationship across multiple scans of the same patient.

### Conduction Velocity and LGE-CMRI Defined Fibrosis

Lower local CV were observed in LA regions with increased fibrotic change as defined by the higher extent of gadolinium enhancement. The conduction delay can be explained by several underlying pathophysiological mechanisms including gap junctional remodeling, Na + channel abnormalities and heterogeneous cell-coupling between myocytes and fibroblasts (Rook et al., 1992; Maleckar et al., 2009; King et al., 2013).

### Measurement Scale

The area subtended by the triads used in the analysis had a clear influence on the resulting beta estimate. In this study, the association is determined using triads of area <80 mm^2^, allowing for a more direct translation and relevance to catheter ablation in clinical practice. At the upper end of this range (70–80 mm^2^), the average maximal edge length of each triad was 15.4 mm (range 5.9–20.0 mm). For comparison, the typical contact area of the ablation electrode is approximately 10 mm^2^ (3.5 mm diameter). Intuitively, averaging over larger areas diffuses the effect of small-scale variations in the quantities of interest and potentially masks the true localized association between them. In the atrium this is crucial, due to the patchy and non-uniform nature of atrial fibrosis (Frustaci et al., 2007). As shown in Figure 2, gadolinium uptake varies on scales as small as 5 mm such as in narrow isthmuses which can promote slow conduction and re-entry (Ciaccio et al., 2008). Fukumoto et al evaluated intensities for each of 20 sectors of the atrial wall in each axial slice. The length of each sector is approximately 20 mm, which is at the upper end of maximal triad edge length used in the present study and correspondingly the larger values of area in Figure 4. Consequently, at larger areas, localized variations in CV would be averaged out leading to a reduction in the magnitude of the association between CV and intensity. This factor may explain the apparent discrepancy between the association (beta estimate) of the present study (β_*1*_: −0.942 m/s/IIR), at smaller spatial scales of areas <80 mm^2^, and that of Fukumoto et al. (β_*1*_: −0.34 m/s/IIR). To explain this, Figure 4 also shows the same area dependency plot expressed in terms of IIR where the red and blue dotted lines mark the beta estimates from our study and that of Fukumoto et al., respectively. The IIR beta estimate of −0.34, found in Fukumoto et al. for persistent AF patients, is of smaller magnitude than the IIR beta estimate of −0.94 reported here, but is consistent with our findings, if measurement area is taken into consideration. This further emphasizes the importance of resolution when quantitatively comparing quantities.

### Reproducibility

While both scans for each patient in the reproducibility sub-study were compared with the same EAM data, our experience suggests that the segmentation and co-registration steps are most prone to the introduction of errors. Importantly, the relationship between CV and NI within each pair of datasets were statistically indistinguishable, confirming the accuracy and reproducibility of our approach.

### Intensity Normalization

One significant contribution of our study is a comparison between the IIR and NI metrics for the qualitative assessment of independently acquired MR images.

By its definition, IIR accounts for underlying shifts in the intensity spectrum, while NI includes the standard deviation of the blood pool to account for inter- and intra-patient differences and accordingly for differences in the dynamic range of the images. Consequently, NI led to a more robust statistical model for elucidating the relationship between CV and intensity, compared with IIR, as well as improved reproducibility of the patient-specific association. In particular, the 95% confidence intervals on the intercept and slope estimates for IIR, shown in Figure 5B, were generally larger than the corresponding confidence intervals for NI in Figure 5A. Reproducibility of patient-specific slopes was observed for NI, but not for IIR.

### Conduction Velocity Restitution

No statistically significant effects due to CL were observed, indicating no identifiable CV restitution.

### Limitations

In order to obtain high fidelity electrograms, electroanatomical data were limited to the posterior LA, which was anatomically consistent, conducive to placement of the AFocusII mapping catheter tangential to the endocardial surface, and contained a predilection of fibrosis. Sampling from the posterior wall may have potentially introduced sampling bias. Future studies should incorporate contact sensing catheters to increase robustness of the data collection protocol.

This study investigated a persistent AF cohort which has been shown to have more extensive structural and electrical remodeling compared to paroxysmal AF patients. Future studies should include a more heterogeneous group of patients.

The image MR images used in this study were manually segmented to delineate the epicardial wall of the left atrium. As such, errors may have been introduced during the segmentation process.

Image intensity ratio is defined as the ratio of the wall intensity of the sector to the blood pool mean (Khurram et al., 2014). However, in this study, IIR was evaluated point-wise as the ratio of the epicardial wall intensity and the mean blood intensity.

## Conclusion

Higher LA intensities correspond to lower local myocardial conduction velocities. The scale of measurement of CV and LA wall intensity is crucial in accurately quantifying this relationship, which is found to be of a higher magnitude than previously reported^1^. Importantly, NI, but not IIR, accounts for changes in the dynamic range of LGE-CMRI and improves the quantitative reproducibility of the relationship. Evaluation of the LA substrate with the use of normalized intensity from LGE-CMRI can be potentially used as a minimally invasive tool to predict atrial myocardial conduction properties.

## Data Availability Statement

The raw data supporting the conclusions of this article will be made available by the authors, without undue reservation.

## Ethics Statement

The studies involving human participants were reviewed and approved by Local Research and Ethics Committee for Imperial College Healthcare NHS Trust. The patients/participants provided their written informed consent to participate in this study.

## Author Contributions

RA, NQ, and CC conceived and planned the experiments. RA developed and implemented the algorithms. NQ obtained the clinical data and segmented the MRIs. SL and CC developed the statistical model and performed the statistical analysis. CR and SK contributed to the interpretation of the results. PL, JT, and NP helped supervise the project. All authors provided critical feedback and helped shape the research, analysis and manuscript.

## Conflict of Interest

SK was employed by the company Abbott United States. The remaining authors declare that the research was conducted in the absence of any commercial or financial relationships that could be construed as a potential conflict of interest.

## Abbreviations

CL: Cycle length
CV: Conduction velocity
EAM: Electro anatomical mapping
IIR: Image Intensity Ratio
LGE-CMRI: Late gadolinium enhanced cardiac MRI
NI: Normalized Intensity.
