# Supplementary material for: Left Atrial Enhancement Correlates With Myocardial Conduction Velocity in Patients With Persistent Atrial Fibrillation
Source: Front Physiol. 2020 Nov 12;11:570203. doi: 10.3389/fphys.2020.570203 (PMC7693630; doi:10.3389/fphys.2020.570203)
Supplement: Supplementary file 1 [file Table_1.DOCX]

Figures:


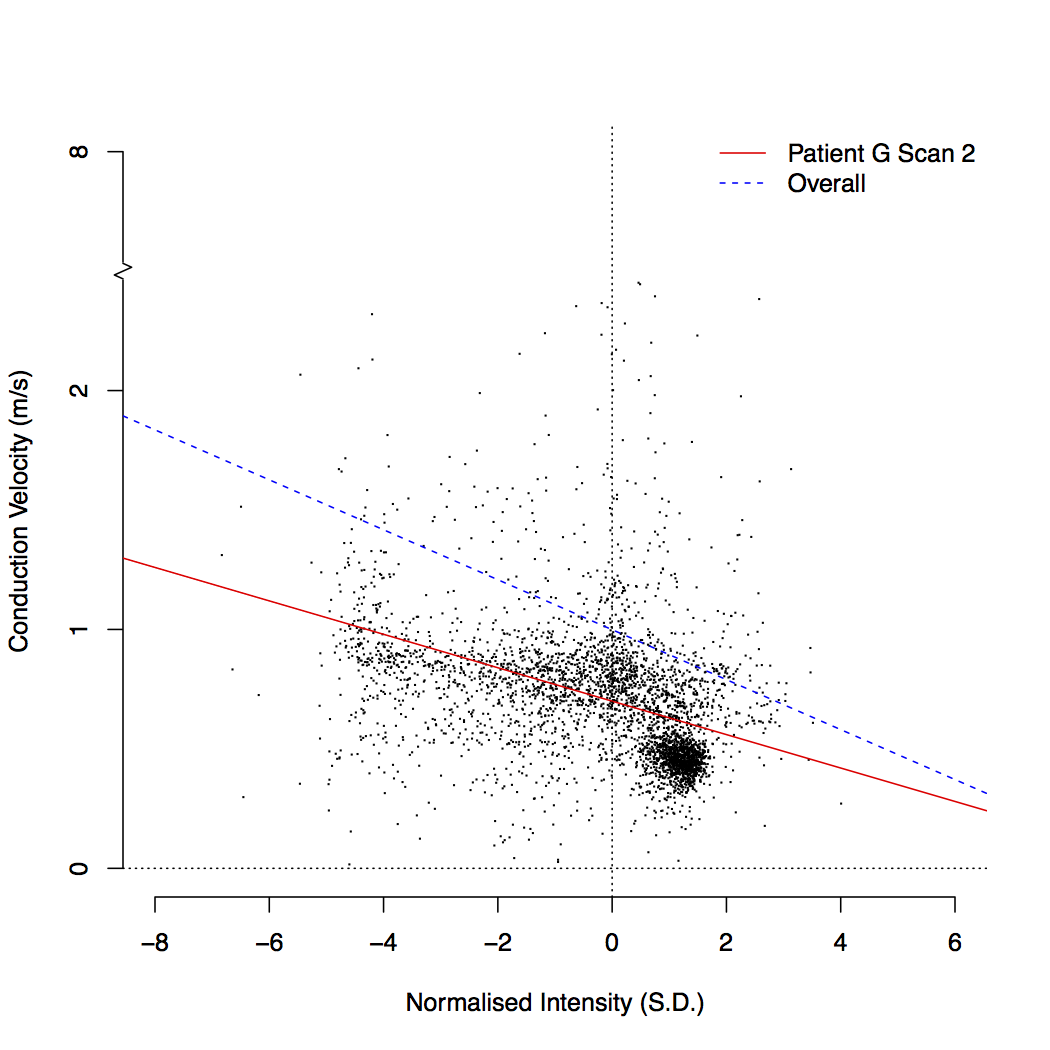


Figure S1: Association of conduction velocity with normalised intensity for all kernels from Patient G, scan 2. Each dot corresponds to a triad formed from three concurrently recorded electrograms within a kernel. Red line shows patient-specific association. Blue dotted line shows overall association.

| **Patient** | **CV (mean, m/s)** | **CV (SD, m/s)** | **NI (mean)** | **NI (SD)** |
| --- | --- | --- | --- | --- |
| A | 0.86 | 0.231 | 0.75 | 0.72 |
| B | 0.67 | 0.08 | 1.89 | 1.19 |
| C | 0.53 | 0.24 | 0.38 | 0.34 |
| D | 0.7 | 0.21 | 1.72 | 0.55 |
| E | 0.67 | 0.30 | 1.16 | 0.90 |
| F | 0.84 | 0.34 | 0.67 | 1.66 |
| G1 | 0.74 | 0.48 | 0.14 | 0.56 |
| G2 |  |  | -0.11 | 1.81 |
| H1 | 0.75 | 0.52 | 0.88 | 1.16 |
| H2 |  |  | 1.31 | 0.50 |

Table S1: Summary statistics for local CV and NI across patients.

| Patient  [# of LGE-MR] | Pacing Location | Cycle Length (ms) | | | | |
| --- | --- | --- | --- | --- | --- | --- |
|  |  | 600 | 400 | 350 | 300 | 250 |
| A [1] | CS | 100 | 100 | 0 | 100 | 100 |
|  | ROOF | 100 | 100 | 0 | 100 | 100 |
| B [1] | CS | 80 | 80 | 80 | 80 | 80 |
|  | LAA | 60 | 60 | 60 | 60 | 60 |
| C [1] | CS | 100 | 100 | 100 | 100 | 100 |
|  | ROOF | 100 | 80 | 60 | 80 | 80 |
| D [1] | CS | 80 | 80 | 80 | 80 | 40 |
|  | LAA | 60 | 60 | 80 | 80 | 0 |
| E [1] | CS | 400 | - | - | 380 | - |
| F [1] | CS | 360 | - | - | 340 | - |
| G [2] | CS | 520 | - | - | 520 | - |
| H [2] | CS | 320 | - | - | 320 | - |

Table S2: Summary of pacing locations and number of electrograms collected at each cycle length for each patient. The number enclosed in the bracket indicates the number of pre-ablation LGE-MRIs for that patient.
